# High platelet distribution width is an independent risk factor of postoperative pneumonia in patients with type A acute aortic dissection

**DOI:** 10.3389/fcvm.2022.984693

**Published:** 2022-09-15

**Authors:** Xi Xie, Danyang Yan, Xin Liu, Yanjie Wang, Ying Deng, Run Yao, Ning Li

**Affiliations:** ^1^Department of Blood Transfusion, National Clinical Research Center for Geriatric Disorders, Xiangya Hospital, Clinical Transfusion Research Center, Central South University, Changsha, China; ^2^Department of Clinical Pharmacology, Xiangya Hospital, Central South University, Changsha, China; ^3^Ningxiang People's Hospital Affiliated to Hunan University of Traditional Chinese Medicine, Ningxiang, China

**Keywords:** platelet distribution width, postoperative pneumonia, type A acute aortic dissection, risk factor, platelet activation

## Abstract

**Background:**

Platelet distribution width (PDW), as a widely applied and reliable marker of platelet activation, was associated with adverse outcomes in cardiovascular diseases. However, there is little literature on the relationship between PDW and postoperative pneumonia in patients with type A acute aortic dissection (AAAD).

**Methods:**

In this retrospective cohort study, we collected consecutive patients who underwent emergency surgery for AAAD at Xiangya Hospital of Central South University from January 1, 2014 and June 30, 2020. Patients were divided into three tertiles on the basis of the PDW. The independent effect of the PDW on postoperative pneumonia was evaluated using multivariate logistic regression analysis, and smooth curve fitting was performed to visualize the linear relationship between PDW and the risk of postoperative pneumonia in patients with AAAD.

**Results:**

A total of 210 patients with AAAD were enrolled and the overall incidence of postoperative pneumonia was 25.24% (*n* = 53). Multivariate logistic regression revealed that PDW was positively associated with the risk of postoperative pneumonia (OR: 1.07, 95% CI: 1.02–1.13, *P* < 0.05) after adjusting the confounders. Compared with the lowest PDW tertile, the risk of postoperative pneumonia increased by 1.21-fold in the medium PDW tertile (OR: 2.21, 95% CI: 0.73–6.72) and by 3.16-fold in the highest PDW tertile (OR: 4.16, 95% CI: 1.40–12.33). A straight-line relationship was observed between PDW and postoperative pneumonia risk in smoothing spline fitting.

**Conclusion:**

Our findings indicate that high PDW is an independent risk factor of postoperative pneumonia in patients with AAAD. Preoperative PDW may serve as an available indicator of pneumonia, which helps identify AAAD patients with a high risk of postoperative pneumonia.

## Introduction

Type A acute aortic dissection (AAAD), characterized by intimal tear and propagation of the dissection between the media and intima layers of the ascending aorta, is a lethal cardiovascular emergency with an estimated 73% overall case-fatality rate and 49% pre-hospital mortality (1). A good operative strategy and precise emergency surgery can increase the survival rate of AAAD patients, which accompanied multiple postoperative complications (2, 3). Postoperative pneumonia is the main infectious complication following cardiac surgery and associated with significant increases in mortality and health care costs (4). Predictive biomarkers for identifying the increased risk of developing postoperative pneumonia in AAAD patients are important for both risk stratification and early treatment strategies.

Platelets play a vital role in the pathogenesis of thrombotic events and atherosclerotic plaque rupture; thus, the application of platelets parameters in risk stratification for cardiac patients was verified in numerous studies (5–7). Previously, our team reported that low platelet count was a risk factor of postoperative pneumonia in patients with AAAD (2). We speculated the platelet consumption possibly due to platelet activation, which was related to arterial dissection occurrence and inflammation response. Platelet distribution width (PDW) is a cheap and generally available biomarker of platelet activation (8), which allows for predicting adverse events in a variety of diseases. Increased levels of PDW were presumed to be associated with poor prognosis in acute myocardial infarction treated with primary percutaneous coronary intervention (5, 6, 9), coronary artery disease (7, 10) and various cancers (11–13). PDW had been disclosed to be a more specific indicator of platelet reactivity than mean platelet volume (7, 14), since it didn't increase during simple platelet swelling. However, there is less data regarding the association between PDW and postoperative pneumonia in patients with AAAD.

Therefore, we aimed to explore the relationship between PDW with the risk of postoperative pneumonia in patients with AAAD, and to provide a reference for further explaining the role of platelet activation in postoperative pneumonia in AAAD patients.

## Materials and methods

### Patient selection

In this retrospective cohort study, we identified patients with AAAD who underwent surgery within 48 h of admission to Xiangya Hospital of Central South University between January 1, 2014 and June 30, 2020. Patients with aches or other related symptoms that occurred within 2 weeks before admission, as well as AAAD-related computed tomography and echocardiography findings, were diagnosed as AAAD (15). Stanford type A (DeBakey type I and type II) dissection involved the ascending aorta with or without aortic arch. Eligible participants were enrolled in the final analysis if they met the following criteria. Inclusion criteria: (a) diagnosed as AAAD; (b) treated with emergency cardiac surgery within 48 h; (c) age ≥ 18 years. Exclusion criteria: (a) preoperative pneumonia or multiple organ failure; (b) death in operation or within 48 h after surgery; (c) no PDW data at admission (3). The procedures used in this study complied with the Declaration of Helsinki. The Medical Ethics Committee of Xiangya Hospital of Central South University approved this study and waived the need for informed consent from individual patients owing to the retrospective nature of the study (Ethical Number: 2019010038).

### Data collection

We collected patients' demographic, laboratory, and clinical data from medical records and checked by two reviewers. Demographic data included age and sex. Medical history included smoking; alcohol consumption; transfusion history; Marfan syndrome; hypertension; diabetes mellitus; chronic obstructive pulmonary diseases; asthma; immunodeficiency; hemopericardium; chronic renal failure; cerebrovascular disease and coronary artery disease. Laboratory data included hemoglobin; neutrophils; lymphocyte; platelet count; mean platelet volume; PDW; hematocrit; red blood cell distribution width; prothrombin time; international normalized ratio; activated partial thromboplastin time and D-Dimer. Hospital management included surgery type; cardiopulmonary bypass time; duration of ventilator; autologous blood transfusion (≥500 mL); blood type and blood product transfusion [red blood cells (RBCs); plasma; cryoprecipitate; and platelet]; antibiotics; hospital and intensive care unit (ICU) stay. The duration of ventilator time for patients was calculated from the time of their intraoperative tracheal intubation to the time of their ventilator discontinuation. All patients were treated with antibiotics for anti-inflammatory treatment during and after operation.

PDW, defined as the coefficient of variation for the measured platelet volume size, was obtained from routine blood tests. For the measurement of PDW, the first venous blood samples taken at the time of patient admission were collected in K2-ethylenediaminetetraacetic acid tubes and analyzed by the Sysmex XN-20A1 analyzer [Beckman Coulter Trading (China) Co., Ltd. Shanghai, China]. The laboratory normal reference for PDW was <17.2%. All laboratory data were derived from the routine blood tests, and the first venous blood samples of patients were collected when they were admitted to the hospital.

### Endpoint

The endpoint was the development of pneumonia after surgery based on the US Centers for Disease Control criteria (16). Two or more serial chest radiographs with at least one of the following (one radiograph is sufficient for patients with no underlying pulmonary or cardiac disease): (a) New or progressive and persistent infiltrates, (b) consolidation, (c) cavitation; and at least one of the following: (a) fever (>38°C) with no other recognized cause, (b) leukopenia (white cell count < 4 × 10^9^) or leukocytosis (white cell count > 12 × 10^9^), (c) for adults > 70 years old, altered mental status with no other recognized cause; and at least two of the following: (a) new onset of purulent sputum or a change in character of the sputum, or increased respiratory secretions, or increased suctioning requirements, (b) new onset or worsening cough, or dyspnea, or tachypnea, (c) rales or bronchial breath sounds, (d) worsening gas exchange (hypoxemia, increased oxygen requirement, increased ventilator demand). Postoperative pneumonia in this study included hospital-acquired and ventilator-associated pneumonia.

### Statistical analysis

Continuous variables were presented as means ± standard deviations or medians with quartile ranges as appropriate. Categorical variables were presented as frequencies and percentages. Continuous variables were compared using the One-Way ANOVA (for a normal distribution) or Kruskal-Wallis H test (for a skewed distribution). The χ^2^ test was used to compare categorical variables. For missing data on D-Dimer (13.33%); activated partial thromboplastin time (0.48%); cardiopulmonary bypass time (0.48%); duration of ventilator (5.24%); autologous blood transfusion (0.48%) and ICU stay (0.48%), the medians were imputed.

Based on the PDW, the patients were divided into three tertiles: the lowest; medium; and highest PDW. The baseline characteristics of the three groups were compared. A univariate analysis was used to evaluate the associations between the variables and postoperative pneumonia. We evaluated the independent effect of PDW on postoperative pneumonia in patients with AAAD using three multiple logistic regression models and two groups based on the reference range. No variables were adjusted in Model I, while age and sex were adjusted in Model II. We selected age, sex and potential confounders (neutrophils; hematocrit; duration of ventilator; RBCs; plasma; cryoprecipitate; hospital and ICU stay) on the basis of their associations with postoperative pneumonia (*P*-value < 0.10) or a change in effect estimate of more than 10% in Model III (17). There was no multicollinearity between these variables. Smooth curve fitting was performed to visualize the linear relationship between PDW and the risk of postoperative pneumonia in patients with AAAD.

All statistical analyses were performed with R (http://www.R-project.org, The R Foundation) and EmpowerStats software (http://www.empowerstats.com, X&Y Solutions, Inc, Boston, MA, USA). A two-tailed *P*-value < 0.05 was considered significant.

## Results

### Participants selection

**Figure 1:**
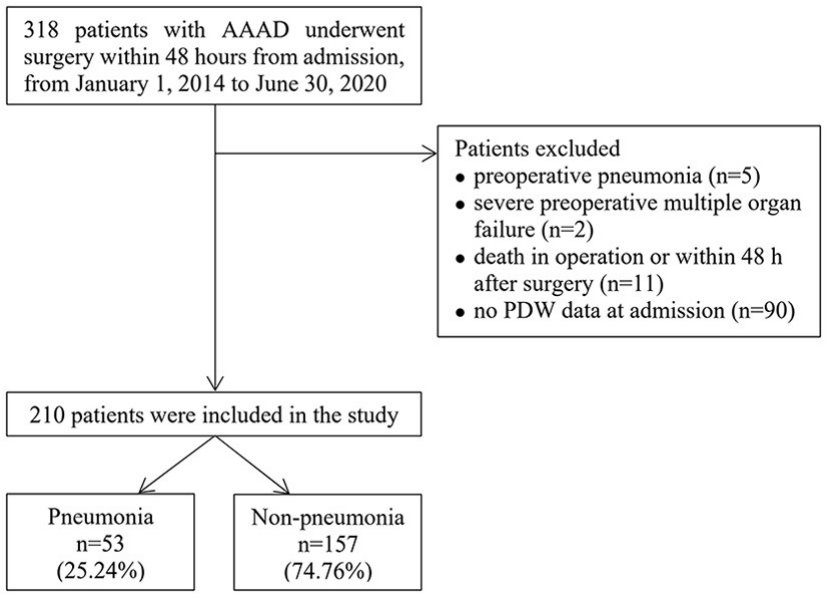
Flow chart of the study.

We identified 318 patients with AAAD who underwent surgery within 48 h of admission. One hundred and eight patients were excluded for the following reasons: preoperative pneumonia (*n* = 5); severe preoperative multiple organ failure (*n* = 2); death in operation or within 48 h after surgery (*n* = 11); no PDW data at admission (*n* = 90). Therefore, a total of 210 patients were enrolled in the final analysis (Figure 1), among which 25.24% (53/210) developed postoperative pneumonia.

### Baseline characteristics

The baseline characteristics are presented by PDW tertiles in Table 1. Intertertiles comparisons showed that the highest incidence (35.62%) of postoperative pneumonia was in patients with the highest PDW tertile (T3: 17.40–19.80%; *P* < 0.01). There were significant differences in Marfan syndrome (*P* = 0.02), chronic renal failure (*P* < 0.01), and coronary artery disease (*P* = 0.01) among the groups. Compared with T1 group (PDW: 14.80–16.70%), the patients in other groups [T2 (PDW: 16.80–17.30%) and T3 (17.40–19.80%)] had significantly lower lymphocyte (*P* = 0.02) and platelet count (*P* < 0.01), longer ICU stay (*P* < 0.05), higher neutrophils and D-Dimer (*P* < 0.01). Moreover, no statistically significant differences were found in the other variables among the groups.

**Table 1:** Baseline characteristics of AAAD patients by PDW tertiles.

**Variables**	**PDW (%) tertiles**			***P*-value**
	**T1 (14.80–16.70)**	**T2 (16.80–17.30)**	**T3 (17.40–19.80)**	
	***n* = 64**	***n* = 73**	***n* = 73**	
**Demographic data**				
Age (years)	47.25 ± 12.73	51.77 ± 10.94	50.45 ± 10.11	0.06
Gender (female)	20 (31.25%)	22 (30.14%)	15 (20.55%)	0.29
**Medical history**				
Smoking	32 (50.00%)	33 (45.21%)	36 (49.32%)	0.83
Alcohol consumption	26 (40.62%)	27 (36.99%)	29 (39.73%)	0.90
Transfusion	2 (3.12%)	1 (1.39%)	1 (1.37%)	0.70
Marfan syndrome	7 (10.94%)	2 (2.74%)	1 (1.37%)	0.02^*^
Hypertension	39 (60.94%)	54 (73.97%)	56 (76.71%)	0.10
Diabetes mellitus	3 (4.69%)	5 (6.85%)	4 (5.48%)	0.86
COPD	1 (1.56%)	4 (5.48%)	2 (2.74%)	0.42
Asthma	0 (0.00%)	1 (1.37%)	0 (0.00%)	0.39
Immunodeficiency	0 (0.00%)	0 (0.00%)	2 (2.74%)	0.15
Hemopericardium	11 (17.19%)	9 (12.33%)	11 (15.07%)	0.72
Chronic renal failure	0 (0.00%)	1 (1.37%)	8 (10.96%)	<0.01^*^
Cerebrovascular disease	2 (3.12%)	1 (1.37%)	4 (5.48%)	0.38
Coronary artery disease	5 (7.81%)	12 (16.44%)	2 (2.74%)	0.01^*^
**Laboratory data**				
Hb (g/L)	120.94 ± 19.56	123.12 ± 21.83	126.22 ± 25.06	0.38
Neutrophils (10^9^/L)	7.81 ± 3.29	10.24 ± 3.54	10.71 ± 4.19	<0.01^*^
lymphocyte (10^9^/L)	1.32 ± 0.56	1.12 ± 0.58)	1.05 ± 0.62	0.02^*^
Platelet count (10^9^/L)	212.72 ± 80.58	161.96 ± 75.02	155.15 ± 60.27	<0.01^*^
MPV (fL)	8.60 ± 1.13	9.06 ± 1.31	9.01 ± 1.44	0.09
hematocrit (%)	36.07 ± 5.46	36.91 ± 6.38	37.76 ± 7.23	0.31
RDW (%)	13.97 ± 1.07	14.16 ± 1.66	14.15 ± 1.79	0.72
PT (s)	13.94 ± 1.45	14.49 ± 1.96	14.76 ± 3.43	0.14
INR	1.12 ± 0.29	1.15 ± 0.16	1.19 ± 0.30	0.29
APTT (s)	35.55 (31.53–39.57)	33.90 (30.30–37.10)	34.50 (31.30–39.00)	0.27
D-Dimer (mg/L)	1.40 (0.71–1.60)	1.60 (0.94–2.50)	1.81 (1.45–2.25)	<0.01^*^
**Hospital management**				
Surgery type				0.58
AAR + TAR (TAVR) + FET	31 (48.44%)	37 (50.68%)	41 (56.16%)	
Bentall + TAR (TAVR) + FET	15 (23.44%)	12 (16.44%)	11 (15.07%)	
David + TAVR + FET	4 (6.25%)	3 (4.11%)	1 (1.37%)	
Combine others	14 (21.88%)	21 (28.77%)	20 (27.40%)	
CPB time (min)	178.00 (163.00–195.00)	181.00 (156.00–217.00)	178.00 (162.00–208.00)	0.69
Duration of ventilator (h)	33.00 (23.00–73.75)	45.00 (24.00–74.00)	45.00 (24.00–72.00)	0.45
Autologous blood transfusion (≥500 ml)	18 (28.12%)	30 (41.67%)	35 (47.95%)	0.06
Blood type				0.50
A	20 (31.25%)	24 (32.88%)	22 (30.14%)	
B	7 (10.94%)	13 (17.81%)	15 (20.55%)	
O	30 (46.88%)	33 (45.21%)	28 (38.36%)	
AB	7 (10.94%)	3 (4.11%)	8 (10.96%)	
RBCs (unit)	6.50 (3.88–9.00)	7.50 (3.00–12.50)	7.00 (2.00–14.50)	0.87
Plasma (unit)	8.45 (5.22–14.00)	9.50 (4.70–18.00)	10.50 (6.00–18.00)	0.14
Cryoprecipitate (therapeutic dose)	1.00 (0.00–1.00)	1.00 (0.00–2.00)	1.00 (1.00–2.00)	0.13
Platelet (therapeutic dose)	1.00 (0.00–2.00)	1.00 (0.00–2.00)	1.00 (0.00–2.00)	0.67
Antibiotics				0.77
Quinolone	1 (1.56%)	2 (2.74%)	1 (1.37%)	
Penicillin	59 (92.19%)	66 (90.41%)	65 (89.04%)	
Cephalosporin	4 (6.25%)	4 (5.48%)	7 (9.59%)	
Aminoglycoside	0 (0.00%)	1 (1.37%)	0 (0.00%)	
Hospital stay (d)	16.00 (12.00–18.25)	15.00 (11.00–19.00)	16.00 (12.00–19.00)	0.94
ICU stay (d)	4.00 (3.00–6.00)	6.00 (4.00–10.00)	6.00 (4.00–10.00)	<0.05^*^
Postoperative pneumonia	8 (12.50%)	19 (26.03%)	26 (35.62%)	<0.01^*^

Results are expressed as mean ± SD, median (Q1–Q3) or *n* (%).

* *P*-value < 0.05; COPD, chronic obstructive pulmonary diseases; Hb, hemoglobin; MPV, mean platelet volume; PDW, platelet distribution width; RDW, red blood cell distribution width; PT, prothrombin time; INR, international normalized ratio; APTT, activated partial thromboplastin time; CPB, cardiopulmonary bypass; AAR, ascending aorta replacement; TAR, total arch replacement; TAVR, total aortic vascular replacement; FET, frozen elephant trunk; RBCs, red blood cells; ICU, intensive care unit.

### Univariate analysis of postoperative pneumonia

**Figure 2:**
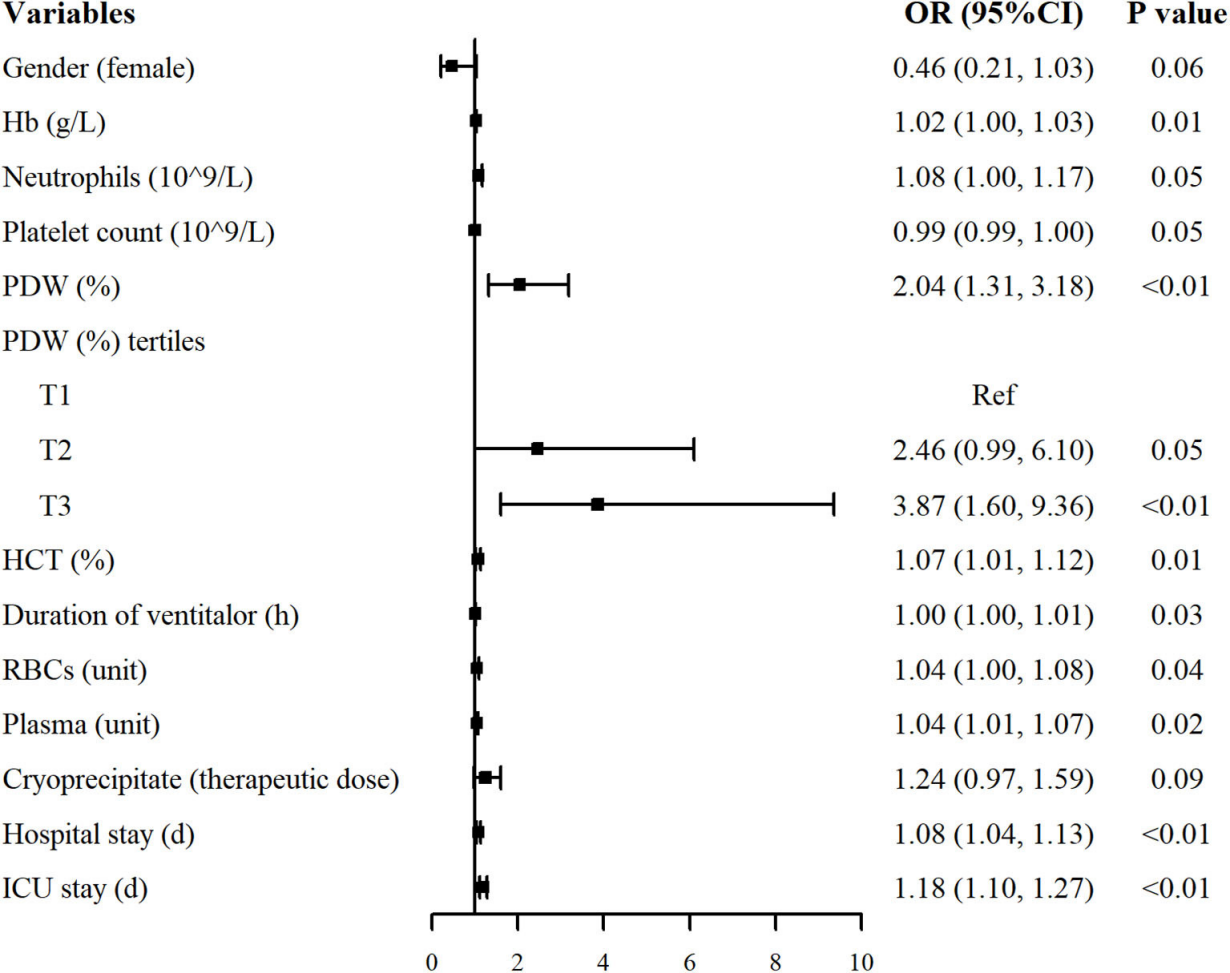
Univariate analysis for postoperative pneumonia in patients with AAAD. Only variables associated with postoperative pneumonia were listed (*P* < 0.10). OR, odds ratio; CI, confidence interval.

The results of the univariate analysis associated with the occurrence of postoperative pneumonia are shown in Figure 2. PDW and PDW tertiles were positively associated with the risk of postoperative pneumonia. Moreover, hemoglobin [Odds ratio (OR): 1.02, 95% confidence interval (CI): 1.00–1.03], neutrophils (OR: 1.08, 95% CI: 1.00–1.17), hematocrit (OR: 1.07, 95% CI: 1.01–1.12), duration of ventilator (OR: 1.00, 95% CI: 1.00–1.01), RBCs (OR: 1.04, 95% CI: 1.00–1.08), plasma (OR: 1.04, 95% CI: 1.01–1.07), hospital stay (OR: 1.08, 95% CI: 1.04–1.13) and ICU stay (OR: 1.18, 95% CI: 1.10–1.27) were positively correlated with the risk of postoperative pneumonia. Other variables were not found to be significantly associated with the risk of postoperative pneumonia (Supplementary Table 1).

### Association of the PDW with postoperative pneumonia

In this study, we constructed three multiple logistic regression models to analyze the independent effects of the PDW on the risk of postoperative pneumonia (Table 2). In non-adjusted model I, the PDW showed a positive correlation with postoperative pneumonia (OR: 1.07, 95% CI: 1.03–1.12). Thus, a rise of 0.1% in the PDW was associated with a 7% increase in the risk of pneumonia. Similar results were observed in Models II (OR: 1.07, 95% CI: 1.02–1.12), and III (OR: 1.07, 95% CI: 1.02–1.13). When the PDW was used as a dichotomous variable according to laboratory reference, the patients with PDW ≥ 17.2% had a 2.28-fold risk of postoperative pneumonia than PDW <17.2% in Model I (OR: 2.28, 95% CI: 1.21–4.30). After adjusting potential confounders, the results did not change obviously in Model II (OR: 2.17, 95% CI: 1.15–4.13) and Model III (OR: 2.39, 95% CI: 1.13–5.06). For the sensitivity analysis, we handled the PDW as three categorical or continuous variables, the trend of the relationship between PDW and postoperative pneumonia was consistent (*P* < 0.01).

**Table 2:** Multivariable regressions analysis in different models.

	**Model I**	**Model II**	**Model III**
	**OR (95% CI)**	**OR (95% CI)**	**OR (95% CI)**
PDW (× 10‰)	1.07 (1.03, 1.12)^*^	1.07 (1.02, 1.12)^*^	1.07 (1.02, 1.13)^*^
**PDW (× 10**‰**) categorical**			
<17.2	Ref	Ref	Ref
≥17.2	2.28 (1.21, 4.30)^*^	2.17 (1.15, 4.13)^*^	2.39 (1.13, 5.06)^*^
**PDW (× 10**‰**) tertiles**			
T1 (14.80–16.70)	Ref	Ref	Ref
T2 (16.80–17.30)	2.46 (0.99, 6.10)	2.54 (1.01, 6.40)^*^	2.21 (0.73, 6.72)
T3 (17.40–19.80)	3.87 (1.60, 9.36)^*^	3.77 (1.53, 9.24)^*^	4.16 (1.40, 12.33)^*^
*P*-value for trend	<0.01	<0.01	<0.01

OR, odds ratio; CI, confidence interval.

* *P-*value < 0.05. Model I adjust for: none. Model II adjust for: age; gender. Model III adjust for: age; gender; neutrophils; hematocrit; duration of ventilator; RBCs; plasma; cryoprecipitate; hospital and ICU stay.

### Linear relationship between the PDW and risk of postoperative pneumonia

**Figure 3:**
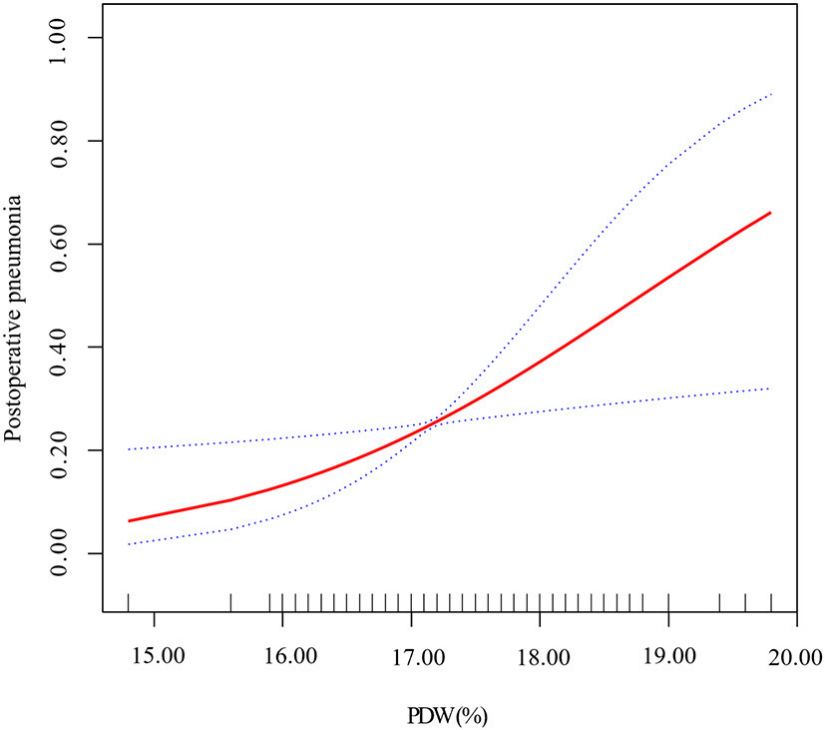
Smoothing spline fitting curve. Adjusted variables: age; gender; neutrophils; hematocrit; duration of ventilator; RBCs; plasma; cryoprecipitate; hospital and ICU stay. The linear plots are displayed with red dotted lines, and the blue dotted lines represent 95% CI.

We applied smoothing spline fitting after adjusting covariates to visualize the relationship between PDW and postoperative pneumonia risk in patients with AAAD. A straight-line relationship between PDW and postoperative pneumonia was observed, which was exactly consistent with the results of multiple logistic regression (Figure 3).

## Discussion

In this study, we confirmed a straight-line relationship between admission PDW and postoperative pneumonia in patients with AAAD and demonstrated that high PDW was significantly associated with an increased risk of postoperative pneumonia in patients with AAAD after adjustment for potential confounders.

PDW, routinely measured by automated cell counters, has emerged as a relatively reliable marker of thrombopoiesis and platelet function (18). PDW is used to reflect the variability in platelet size and detect fractions of larger platelets that are more active, both enzymatically and metabolically (8). In a predictive model for AAAD, Wang et.al (19) displayed that PDW was an independent factor in predicting in-hospital death (hazard ratio: 3.755, 95% CI: 1.436–9.815, *P* < 0.001). A retrospective cohort found that high PDW values (≥16.31%) were independently associated with higher risk of all-cause (hazard ratio: 1.49, 95% CI: 1.15–6.82) and cardiovascular deaths (hazard ratio: 2.26, 95% CI: 1.44–3.63) in hemodialysis patients (20). Regarding to acute myocardial infarction treated with primary percutaneous coronary intervention, Rechciński et al. (6) observed higher PDW values (≥16 fL) correlated with a higher mortality rate when compared with PDW < 16 fL (17.4 vs. 6.3%, *P* = 0.001) in a sample of 538 patients. Celik et al. (5) reported the odds of in-hospital major adverse cardiovascular events increased by 22.3% per unit increase in PDW. Likewise, a retrospective cohort of 679 participants concluded that high PDW level was an independent risk factor of long-term major adverse cardiovascular events in acute coronary syndrome (OR: 1.218, 95% CI: 11.116–1.328; *P* < 0.001) (21). However, few studies investigated the association between PDW and postoperative pneumonia. A previous study from our group indicated that low platelet count was a risk factor of postoperative pneumonia in AAAD patients, possibly due to platelet activation and high consumption (2). Besides, we found a U-shaped relationship between platelet–lymphocyte ratio and in-hospital mortality in patients with AAAD (22), which also revealed a role for platelets in poor prognosis. In the present study, our findings manifested that PDW was positively correlated with the risk of postoperative pneumonia, supporting our previous conjecture. A 0.1% increase of PDW was associated with a 7% increase in the risk of postoperative pneumonia in patients with AAAD. Furthermore, similar trends were observed whether PDW was handled as three equal groups or dichotomous variables. Compared with the lowest PDW group (T1), the risk of postoperative pneumonia increased by 1.21-fold in the medium PDW group (T2) and by 3.16-fold in the highest PDW group (T3) after adjusting potential confounders. Likewise, compared with the laboratory recommended reference range (PDW < 17.2%), patients with PDW ≥17.2% had a 1.39-fold increased risk of postoperative pneumonia. These findings further illustrate the robustness of our results that higher PDW levels were independently associated with higher risk of postoperative pneumonia in AAAD patients. As an available and inexpensive biomarker, PDW may be a promising predictor of early clinical intervention to prevent postoperative pneumonia in AAAD patients, further reduce the financial burden of patients, and even reduce in-hospital mortality. Also, it may provide a reference for further explaining the role of platelet activation in postoperative pneumonia in AAAD patients.

The underlying mechanism of the relationship between elevated PDW and postoperative pneumonia in patients with AAAD still remains unclear. The increase of PDW usually occurs as a result of platelet activation (23), involving the synthesis of prothrombotic and proinflammatory agents in platelets, morphological alteration, degranulation of alpha-granules and release of highly reactive platelets from stores. It is currently accepted that platelets activation might have a critical role in initiation and progression of post-dissection inflammatory responses. Activated platelets in circulation could release diverse bioactive substances to initiate or amplify inflammation (24, 25). These proinflammatory molecules could exacerbate leukocytes rolling, adhesion and recruitment (26), thereby triggering lung inflammation and deteriorating the state of lung infection. In turn, a diverse array of growth factors and cytokines in the atherosclerotic processes may increase levels of PDW through altering the morphology and reactivity of platelets released from the bone marrow (27, 28). In an AAAD canine model, mean platelet volume/platelet count were significantly higher after AAAD and positively correlated with TNF-α (*r* = 0.826, *P* = 0.011) and IL-6 (*r* = 0.806, *P* = 0.016), which verified the association between the activated platelets and inflammatory cytokines (29). Additionally, experimental and clinical observations indicate that platelets regulate pulmonary microvascular endothelial barrier function and integrity, and influence pathological inflammatory processes at the alveolar-capillary membrane, in the tracheobronchial tree, and in lung vessels (30). Taken together, elevated PDW was entirely reasonable to be associated with postoperative pneumonia in patients with AAAD.

There are several limitations in our study. First, this is a single-center, small sample retrospective study with an inevitable bias. We adjusted the confounders as much as possible in the statistical analysis to control the potential bias, so the results of this study have a certain credibility. Secondly, this was a retrospective study and can only show the association between PDW and postoperative pneumonia. More prospective, multicenter and large-scale studies are needed in the future to validate the relationship between PDW and postoperative pneumonia in patients with AAAD. Thirdly, there is no gold standard for diagnosis of hospital-acquired or ventilator-associated pneumonia, and we could not strictly distinguish hospital-acquired pneumonia from ventilator-associated pneumonia. Finally, information about the dynamic alteration of PDW during hospitalization was not available. Future studies should focus on the changes in PDW with time after surgery and how they are related to clinical outcome in AAAD patients.

## Conclusion

To conclude, high PDW is an independent risk factor of postoperative pneumonia in patients with AAAD in this study. And preoperative PDW may serve as a simple and widely available indicator of pneumonia, which could help identify AAAD patients in high risk of postoperative pneumonia.

## Data availability statement

The raw data supporting the conclusions of this article will be made available by the authors, without undue reservation.

## Ethics statement

The studies involving human participants were reviewed and approved by the Medical Ethics Committee of Xiangya Hospital of Central South University (Ethical Number: 2019010038). Written informed consent for participation was not required for this study in accordance with the national legislation and the institutional requirements.

## Author contributions

XL, YW, and YD collected and assembled data. YD and XX operated the software and statistical analysis. RY, DY, and XX performed the data analysis and interpretation. XX contributed to writing–original draft. NL and RY obtained the funding. All authors contributed to the study concept and design. All authors read and approved the final version of the manuscript.

## Funding

This work was funded by the Youth Fund of Xiangya Hospital, Central South University (Grant/Award No. 2021Q13) and Natural Science Foundation of Hunan Province (Grant/Award Nos. 2022JJ40840 and 2022JJ70076). The funding bodies did not have role in the design of the study, data collection and analysis, nor on the interpretation and dissemination of the results.

## Conflict of interest

The authors declare that the research was conducted in the absence of any commercial or financial relationships that could be construed as a potential conflict of interest.

## Publisher's note

All claims expressed in this article are solely those of the authors and do not necessarily represent those of their affiliated organizations, or those of the publisher, the editors and the reviewers. Any product that may be evaluated in this article, or claim that may be made by its manufacturer, is not guaranteed or endorsed by the publisher.
