# Supplementary material for: High platelet distribution width is an independent risk factor of postoperative pneumonia in patients with type A acute aortic dissection
Source: Front Cardiovasc Med. 2022 Sep 15;9:984693. doi: 10.3389/fcvm.2022.984693 (PMC9521668; doi:10.3389/fcvm.2022.984693)
Supplement: Supplementary file 1 [file Table_1.docx]

Supplementary Material

# Supplementary Table

Table S1. Univariate analysis of variables associated with postoperative pneumonia in patients with AAAD.

| **Variables** | **No pneumonia**  **(n = 157)** | **Pneumonia**  **(n = 53)** | **OR (95% CI)** | **P value** |
| --- | --- | --- | --- | --- |
| Age (years) | 50.09 ± 11.47 | 49.47 ± 11.08 | 1.00 (0.97, 1.02) | 0.73 |
| Gender (female) | 48 (30.57%) | 9 (16.98%) | 0.46 (0.21, 1.03) | 0.06 |
| Transfusion | 3 (1.91%) | 1 (1.92%) | 1.01 (0.10, 9.89) | 0.10 |
| Smoking | 74 (47.13%) | 27 (50.94%) | 1.16 (0.62, 2.17) | 0.63 |
| Alcohol consumption | 60 (38.22%) | 22 (41.51%) | 1.15 (0.61, 2.16) | 0.67 |
| Marfan syndrome | 8 (5.10%) | 2 (3.77%) | 0.73 (0.15, 3.55) | 0.70 |
| Hypertension | 112 (71.34%) | 37 (69.81%) | 0.93 (0.47, 1.84) | 0.83 |
| Diabetes mellitus | 7 (4.46%) | 5 (9.43%) | 2.23 (0.68, 7.36) | 0.18 |
| COPD | 4 (2.55%) | 3 (5.66%) | 2.29 (0.50, 10.61) | 0.29 |
| Asthma | 1 (0.64%) | 0 (0.00%) | 0.00 (0.00, 0.00) | 0.99 |
| Immunodeficiency | 1 (0.64%) | 1 (1.89%) | 3.00 (0.18, 48.82) | 0.44 |
| Hemopericardium | 22 (14.01%) | 9 (16.98%) | 1.26 (0.54, 2.93) | 0.60 |
| Chronic renal failure | 6 (3.82%) | 3 (5.66%) | 1.51 (0.36, 6.26) | 0.57 |
| Cerebrovascular disease | 4 (2.55%) | 3 (5.66%) | 2.29 (0.50, 10.61) | 0.28 |
| Coronary artery disease | 17 (10.83%) | 2 (3.77%) | 0.32 (0.07, 1.45) | 0.12 |
| Hemoglobin (g/L) | 121.30 ± 21.31 | 130.15 ± 24.27 | 1.02 (1.00, 1.03) | 0.01^*^ |
| Neutrophils (10^9^/L) | 9.35 ± 3.83 | 10.60 ± 3.97 | 1.08 (1.00, 1.17) | 0.046^*^ |
| Lymphocyte (10^9^/L) | 1.19 ± 0.61 | 1.07 ± 0.54 | 0.69 (0.39, 1.24) | 0.21 |
| Platelet count (10^9^/L) | 181.22 ± 81.76 | 156.83 ± 52.20 | 0.99 (0.99, 1.00) | 0.045^*^ |
| MPV (fL) | 8.83 ± 1.37 | 9.12 ± 1.13 | 1.18 (0.94, 1.49) | 0.16 |
| PDW (%) | 17.08 ± 0.70 | 17.45 ± 0.73 | 2.04 (1.31, 3.18) | <0.01^*^ |
| PDW (%) tertile |  |  |  | <0.01^*^ |
| T1 | 56 (35.67%) | 8 (15.09%) | Ref |  |
| T2 | 54 (34.39%) | 19 (35.85%) | 2.46 (0.99, 6.10) | 0.05 |
| T3 | 47 (29.94%) | 26 (49.06%) | 3.87 (1.60, 9.36) | <0.01^*^ |
| HCT (%) | 36.31 ± 6.10 | 38.84 ± 7.08 | 1.07 (1.01, 1.12) | 0.01^*^ |
| RDW (%) | 14.10 ± 1.48 | 14.10 ± 1.76 | 1.00 (0.82, 1.23) | 0.97 |
| PT (s) | 14.42 ± 2.57 | 14.41 ± 2.17 | 1.00 (0.88, 1.13) | 0.98 |
| INR | 1.16 ± 0.28 | 1.16 ± 0.17 | 1.00 (0.30, 3.37) | 0.99 |
| APTT (s) | 35.10 (31.00–39.20) | 33.00 (31.20–37.50) | 1.01 (0.99, 1.02) | 0.33 |
| D-Dimer (mg/L) | 1.60 (0.97–2.23) | 1.60 (1.01–2.15) | 1.00 (0.88, 1.13) | 1.00 |
| Surgery type |  |  |  | 0.69 |
| AAR+TAR(TAVR)+FET | 81 (51.59%) | 28 (52.83%) | Ref |  |
| Bentall+TAR(TAVR)+FET | 30 (19.11%) | 8 (15.09%) | 0.77 (0.32, 1.88) | 0.57 |
| David+TAVR+FET | 7 (4.46%) | 1 (1.89%) | 0.41 (0.05, 3.51) | 0.42 |
| Combine others | 39 (24.84%) | 16 (30.19%) | 1.19 (0.58, 2.45) | 0.64 |
| CPB time (min) | 177.00 (158.00–197.00) | 192.00 (169.00–223.00) | 1.00 (1.00, 1.01) | 0.12 |
| Duration of ventilator (h) | 44.00 (23.00–72.00) | 60.00 (30.00–120.00) | 1.00 (1.00–1.01) | 0.03^*^ |
| Ventilator use | 103 (65.61%) | 36 (67.92%) | 1.11 (0.57, 2.16) | 0.76 |
| Autologous blood transfusion (≥500ml) | 60 (38.22%) | 23 (44.23%) | 1.28 (0.68, 2.42) | 0.44 |
| Blood type |  |  |  | 0.86 |
| A | 49 (31.21%) | 17 (32.08%) | Ref |  |
| B | 26 (16.56%) | 9 (16.98%) | 1.00 (0.39, 2.55) | 0.10 |
| O | 67 (42.68%) | 24 (45.28%) | 1.03 (0.50, 2.13) | 0.93 |
| AB | 15 (9.55%) | 3 (5.66%) | 0.58 (0.15, 2.24) | 0.43 |
| RBCs (unit) | 6.50 (3.00–11.00) | 9.00 (4.00–17.00) | 1.04 (1.00, 1.08) | 0.04^*^ |
| Plasma (unit) | 9.30 (4.70–15.40) | 10.50 (7.00–22.00) | 1.04 (1.01, 1.07) | 0.02^*^ |
| Cryoprecipitate (therapeutic dose) | 1.00 (0.00–2.00) | 1.00 (1.00–2.00) | 1.24 (0.97, 1.59) | 0.09 |
| Platelet (therapeutic dose) | 1.00 (0.00–2.00) | 1.00 (1.00–2.00) | 1.15 (0.90, 1.46) | 0.26 |
| Antibiotics |  |  |  | 0.52 |
| quinolone | 2 (1.27%) | 2 (3.77%) | Ref |  |
| penicillin | 144 (91.72%) | 46 (86.79%) | 0.32 (0.04, 2.33) | 0.26 |
| cephalosporin | 10 (6.37%) | 5 (9.43%) | 0.50 (0.05, 4.67) | 0.54 |
| [aminoglycoside](javascript:;) | 1 (0.64%) | 1 (1.37%) | 0.00 (0.00, 0.00) | 0.99 |
| Hospital stay (d) | 15.00 (12.00–18.00) | 19.00 (13.00–28.00) | 1.08 (1.04, 1.13) | <0.01^*^ |
| ICU stay (d) | 5.00 (4.00–7.00) | 9.00 (4.00–13.00) | 1.18 (1.10, 1.27) | <0.01^*^ |

Results are expressed as mean ± SD, median (Q1–Q3) or n (%). *, P value <0.05. OR, odds ratio; CI, confidence interval; COPD, chronic obstructive pulmonary diseases; MPV, mean platelet volume; PDW, platelet distribution width; HCT, hematocrit; RDW, red blood cell distribution width; PT, prothrombin time; INR, international normalized ratio; APTT, activated partial thromboplastin time; CPB, cardiopulmonary bypass; AAR, ascending aorta replacement; TAR, total arch replacement; TAVR, total aortic vascular replacement; FET, frozen elephant trunk; RBCs, red blood cells; ICU, intensive care unit.
